# Empowering faculty to initiate STEM education transformation: Efficacy of a systems thinking approach

**DOI:** 10.1371/journal.pone.0271123

**Published:** 2022-07-25

**Authors:** Stasinos Stavrianeas, Gita Bangera, Claire Bronson, Steven Byers, William Davis, Alyce DeMarais, Ginger Fitzhugh, Nalani Linder, Carrie Liston, Jenny McFarland, Joann Otto, Pamela Pape-Lindstrom, Carol Pollock, C. Gary Reiness, Erika G. Offerdahl

**Affiliations:** 1 Department of Exercise and Health Science, Willamette University, Salem, Oregon, United States of America; 2 RISE Learning Institute, Bellevue College, Bellevue, Washington, United States of America; 3 Viz–spark, Prescott, Arizona, United States of America; 4 Helping Human Systems, Olympia, Washington, United States of America; 5 School of Molecular Biosciences, Washington State University, Pullman, Washington, United States of America; 6 Biology Department, University of Puget Sound, Tacoma, Washington, United States of America; 7 Education Development Center, Waltham, Massachusetts, United States of America; 8 N.P. Linder Consulting, Tacoma, Washington, United States of America; 9 Biology Department, Edmonds College, Lynnwood, Washington, United States of America; 10 Biology Department, Western Washington University, Bellingham, Washington, United States of America; 11 Life Sciences Department, Everett Community College, Everett, Washington, United States of America; 12 Zoology Department, University of British Columbia, Vancouver, British Columbia, Canada; 13 Biology Department, Lewis and Clark College, Portland, Oregon, United States of America; The University of Sydney School of Biological Sciences: The University of Sydney School of Life and Environmental Sciences, AUSTRALIA

## Abstract

Just a decade ago *Vision and Change in Undergraduate Biology Education*: *A Call to Action* was released, catalyzing several initiatives to transform undergraduate life sciences education. Among these was the Partnership for Undergraduate Life Sciences Education (PULSE), a national organization commissioned to increase the adoption of *Vision and Change* recommendations within academic life sciences departments. PULSE activities have been designed based on the recognition that life sciences departments and faculty are embedded within institutions of higher education which, similar to other large organizations, are complex systems composed of multiple, interconnected subsystems. The organizational change research suggests that effecting large-scale changes (e.g., undergraduate STEM education transformation) may be facilitated by applying systems thinking to change efforts. In this paper we introduce the approach of systems thinking as a professional development tool to empower individual STEM faculty to effect department-level transformation. We briefly describe a professional development experience designed to increase life sciences faculty members’ understanding of systems thinking, present evidence that faculty applied a systems thinking approach to initiate department-level change, and discuss the degree to which transformation efforts were perceived to be successful. Though focused on faculty in the life sciences, our findings are broadly transferable to other efforts seeking to effect change in undergraduate STEM education.

## Introduction

Publication of *Vision and Change*: *A Call to Action* [1] was an important milestone for the transformation of undergraduate life sciences education in the United States because it proposed concrete recommendations for inclusive science teaching that reflect decades of research on how people learn. Furthermore, it fueled several national efforts to reform undergraduate STEM teaching and learning through interventions with individual faculty [2], departments and divisions, and even entire institutions of higher education [3]. The Partnership for Undergraduate Life Sciences Education (PULSE, https://pulse-community.org) is one such effort, a national organization focused on empowering faculty to engage in transformation at the department level in all types of higher education institutions (i.e., community colleges, regional comprehensive universities, research universities, private liberal arts colleges). PULSE activities align with the *Vision and Change* (V&C) recommendations that aim to integrate core concepts and competencies throughout the curriculum; focus on student-centered learning; promote a campus-wide commitment to change; and engage the biology community in the implementation of change. The V&C recommendations include incorporation of the five core life science concepts (evolution; pathways and transformations of energy and matter; information flow, exchange, and storage; structure and function; and systems) and six competencies of science (ability to apply the process of science; ability to use quantitative reasoning; ability to use modeling and simulation; ability to tap into the interdisciplinary nature of science; ability to communicate and collaborate with other disciplines; and ability to understand the relationship between science and society). Accordingly, PULSE was created in 2012 through a collaboration among the National Science Foundation, National Institutes of Health/National Institute of General Medical Sciences, and Howard Hughes Medical Institute. PULSE has focused on leveraging change by working at the department level to increase the implementation of V&C recommendations because these are the academic units that control the curriculum [4].

The PULSE organization is a national network of life sciences faculty and administrators that facilitates departmental transformation through three activities: the PULSE Recognition program, the PULSE Ambassadors program, and PULSE Regional workshops. The PULSE Recognition program provides tools for departments to assess and reflect on progress in implementing V&C recommendations and a formal mechanism for departments to receive external recognition of that progress [5, 6]. Academic departments can invite members of the PULSE Ambassadors program for a multi-day campus workshop during which PULSE-trained facilitators lead the entire department through a process to develop a common vision and action plan for enacting change. Finally, PULSE is organized into five Regional Networks in the United States, each of which hosts workshops and timely programming support and evaluation for departments at various stages of transformation. The design of all PULSE activities recognizes that life sciences departments and faculty are embedded within institutions of higher education, which are complex systems composed of multiple, interconnected subsystems [7].

**Fig 1:**
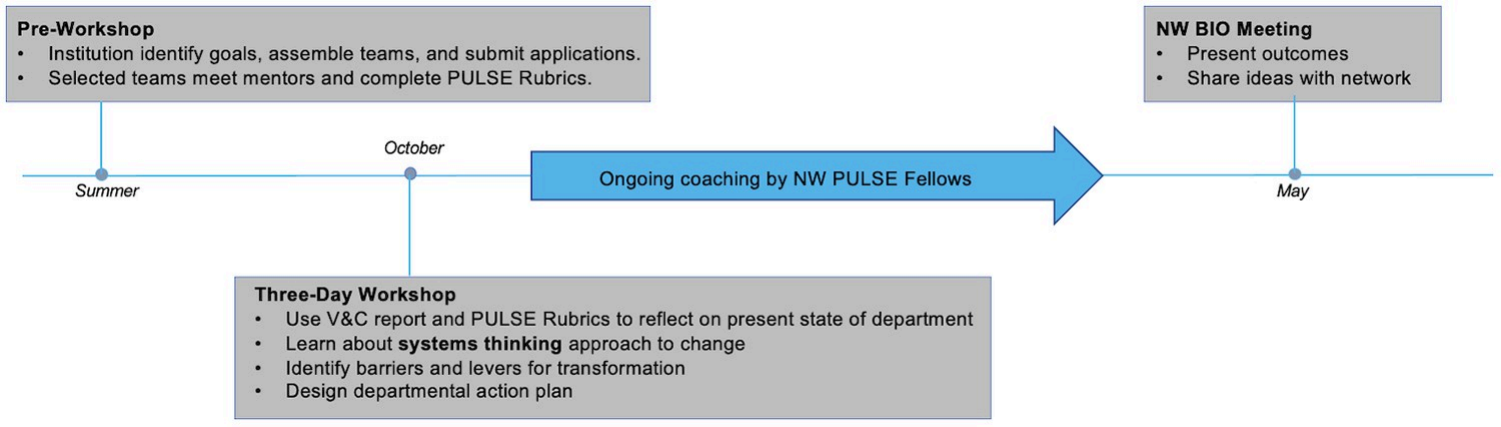
Annual timeline of NW PULSE regional professional development activities.

The leaders of the Northwest PULSE regional network (NW PULSE) represent all types of academic institutions, which is reflective of the diverse education and career paths for students in life sciences. The foundational principle of inclusion guided the development of a professional development experience for life sciences faculty and administrators, especially since we were interested in identifying issues and solutions that transcended institutional silos. The NW PULSE workshop (Fig 1) was a three-day, in-person event; supplemented by ongoing customized mentoring and a culminating networking event, described in greater detail elsewhere [8]. Briefly, life sciences departments across the Northwest (Washington, Oregon, Idaho, Montana, Wyoming, Alaska) and beyond were invited to assemble an institutional team consisting of two to three life sciences faculty and at least one administrator. Applications were reviewed by the NW PULSE Fellows and teams were selected based on their demonstrated capacity for strategic planning, readiness and need for departmental transformation, and geographical and institutional diversity.

**Fig 2:**
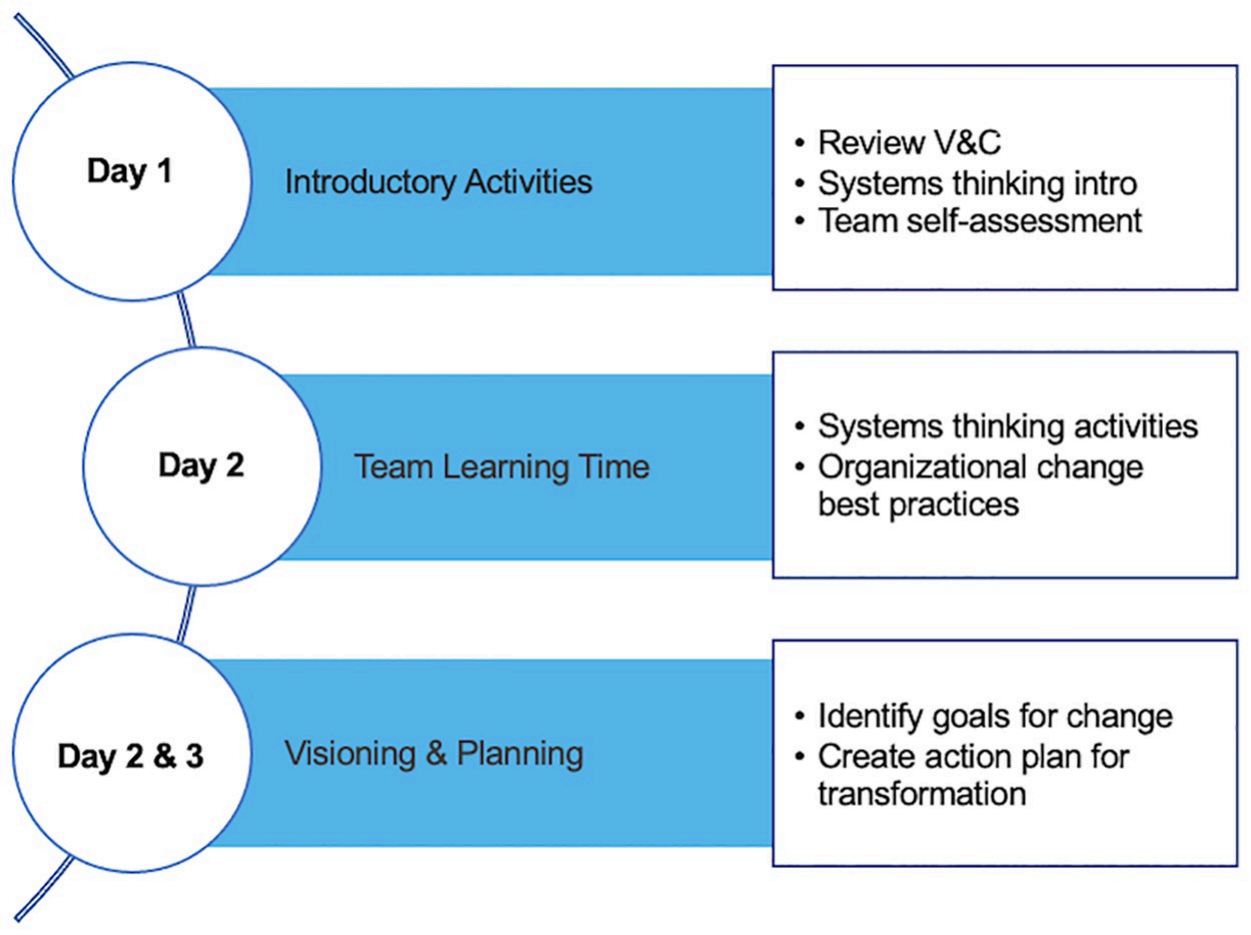
Overview of activities during the three-day NW PULSE workshop.

Prior to each of the three-day workshops, the selected teams completed a self-assessment developed by the PULSE community [6, 9] to diagnose their department’s current status in implementing the recommendations of V&C. Each team was also assigned a mentor from the NW PULSE leadership group (G.B., W.D., A.DeM., J.McF., J.O., P.P.L., C.P., C.G.R., E.G.O., S.S.) who facilitated reflection around departmental culture and vision. During the three-day workshop the teams participated in a series of activities designed to help them learn about the role of individual faculty members in effecting department-level change, in which they were introduced to systems thinking and provided dedicated time to formulate a shared vision and action plan for department transformation (Fig 2).

On Day 1, participants were introduced to the workshop objectives, reviewed the recommendations of V&C, reflected on their departmental self-assessment, and received a brief introduction to systems thinking. These tools were applied during team learning time in Day 2, where institutional teams took inventory of their practices and planned future steps for departmental transformation. These plans were then articulated and presented to the entire group of participants for feedback by Day 3; each institutional team then created a plan of action to be implemented upon return to their home institutions. A more comprehensive description of the professional development activities has been described previously [8].

After the workshop, the mentors remained in contact with their assigned team and provided additional resources and support as needed. The following spring, usually in early May, a representative from each team was expected to attend the annual conference of the Northwest Biology Instructor’s Organization (NW BIO), an organization that seeks to facilitate dialogue among faculty of colleges and universities in the Pacific NW (Washington, Oregon, Idaho, British Columbia) on pedagogical and scientific problems in the field of biology. At that meeting, a networking event was organized for workshop participants during which a representative from each team representative presented a poster outlining initial steps taken and progress made in enacting the action plans formulated during the autumn workshop.

**Fig 3:**
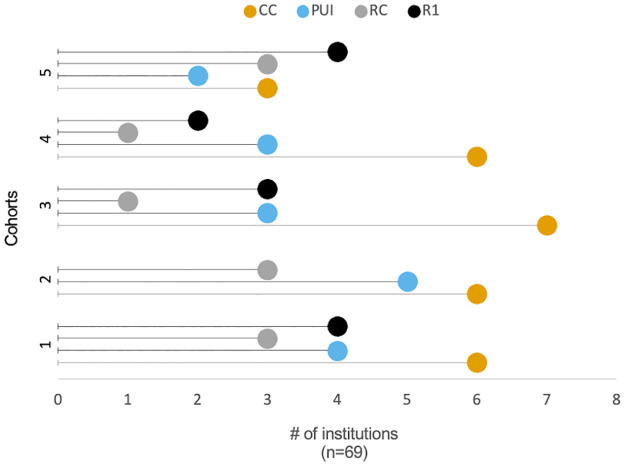
Institutional break down of NW PULSE institutional teams. Five cohorts of institutional teams participated in NW PULSE professional development activities. The diversity of institutions that participated reflects the NW PULSE philosophy of inclusivity and shared interests. (CC: Community Colleges, PUI: Predominantly Undergraduate Institutions, RC: Regional Comprehensive Institutions, R1: Research-intensive Institutions).

In total, five cohorts participated in the NW PULSE regional professional development activities (Fig 3). In all, participants from 69 different institutions attended a workshop; five institutions were represented twice with different teams. Workshop participants (n = 224) were faculty and instructors including 62 administrators (e.g., department chairs, division chairs, deans, associate deans).

The focus of all PULSE activities is catalyzing transformation at the department level. While there are several approaches to bringing about organizational change, research suggests that efforts that empower faculty to effect change within complex institutional systems will be more successful when approached through systems thinking [10–12]. Systems thinking has been defined as “a discipline for seeing wholes” [13] and intentionally seeks to understand the interdependencies among the elements of a system, and for this reason we employed systems thinking as a tool for department-level transformation. When we developed the workshop, using a systems thinking conceptual framework was a novel approach for professional development in the life sciences, and we have described our approach to incorporating systems thinking in departmental transformation elsewhere [8]. Coauthor P.P.L. suggested that a focus on systems thinking would help to support departmental transformation across institution types, and we therefore recruited experts in systems thinking (C.B., S.B., N.L.) to assist in the workshop design and delivery.

In complex systems such as institutions of higher education, cause and effect may not be closely coupled, and feedback is likely to be delayed rather than immediate; consequently, change initiatives may produce unintended consequences [7, 14–16]. As one of the tools available to facilitate change in complex structures such as departments or institutions, systems thinking offers faculty members a means to understand and appreciate the interconnectedness between actions and consequences. The entire process we followed was designed to identify these faculty members interested in instigating change and support them to engage strategies that enhance positive aspects of the system and simultaneously modify those elements that may negatively influence the intended outcome.

Systems thinking addresses how components of a system are interrelated and how they interact over time. That is, it considers organizations in terms of complex, dynamic relationships and interactions among all their parts. If we consider institutions of higher education as systems, we can then employ systems thinking to consider how each of the parts function individually and not assume simple linear cause-and-effect relationships, but also prepare for unintended consequences, time delays and potential feedback loops. The intent of systems thinking is to understand the behavior of a system (i.e., department or institution) as a whole to anticipate more holistically the outcomes of a change initiative which, by necessity, may focus on one or a few elements of the system (e.g., individual faculty members). Application of a systems thinking approach is ideally suited for effective change in complex organizations with many components, where multiple (and often competing) priorities exist. Systems thinking reveals opportunities to leverage or pool resources and achieve short-term successes [7, 11].

It is ultimately through the behavior of multiple individuals within the system (i.e., faculty members, administrators) that transformation will be enacted. Systems thinking facilitates a deep understanding of interactions within the system, including the multiple internal and external factors (e.g., tenure and promotion criteria and policies, resource availability, professional development opportunities, parents, students, state governments, accrediting bodies) that ultimately affect how and what instructors teach [11]. The decisions made by individual faculty members are influenced by the varied contexts within which they work [7, 17]. Each of these contexts represents a different level of influence on a faculty member. It has been suggested that efforts to transform STEM undergraduate teaching and learning will be more successful if approached from a systems framework, anticipating the interactions among players as they relate to the multiple levels of influence within the varied contexts of higher education [11]. Individuals seeking to instigate change will be more adept at explicitly identifying spheres of influence in the system and the potential implications for transformation efforts if they apply systems thinking. For example, influence applied from decisions made by university executive leadership will likely have different implications than transformation efforts exerting influence from the faculty themselves, depending on the characteristics of that particular institutional context.

Influence is applied to leverage change in a variety of ways. The Four Frames model [10], which has its roots in organizational change, has been used more recently to understand common ways in which influence manifests in departmental transformation efforts in undergraduate STEM [17, 18]. In its original inception, the Four Frames model articulated four distinct frames or lenses (structural, human resource, political, symbolic) through which a system can be viewed. Each of the frames provides distinctive insights about the system, its components, and the relationships within and emerging from the system. Transformation efforts are more likely to succeed if influence is leveraged through more than one frame [7, 11]. For example, efforts to transform undergraduate teaching and learning that focus predominantly on providing professional development are operating through the frame of human resources. But professional development alone is not likely to effect long lasting transformation; simultaneously enacting promotion and tenure procedures that reward teaching (structural frame) is more likely to increase the potential for success. Similarly, a department may have accumulated the basic elements of culture to develop a collective identity that values teaching (symbolic frame). But if the department is unable to acquire resources to support engaged student learning (political frame), the instructors will be unable to fully realize their instructional aspirations. The Four Frames model would predict that applying a systems thinking approach to department-level transformation will be evidenced by efforts that identify ways to exert influence through more than one frame.

The NW PULSE regional professional development opportunity included explicit instruction on systems thinking for life sciences faculty and administrators as a mechanism for initiating departmental transformation. If systems thinking were shown to be an effective tool to bring about departmental transformation in the life sciences given the diversity of institutions that participated in our effort, then this approach could be applicable to other life science departments regardless of the breadth of their curriculum or availability of resources. In this retrospective review of outcomes from our work we explore whether this professional development experience with an explicit focus on systems thinking supports transformation in undergraduate life sciences teaching and learning. In doing so, we will answer the following questions:

1. Did the NW PULSE workshop activities increase participants’ self-reported systems thinking skills?
2. Did NW PULSE participants apply systems thinking to initiate transformation at the department level?
3. Were the transformation efforts of the NW PULSE participants successful?

## Methods

### Data collection and analysis

**Fig 4:**
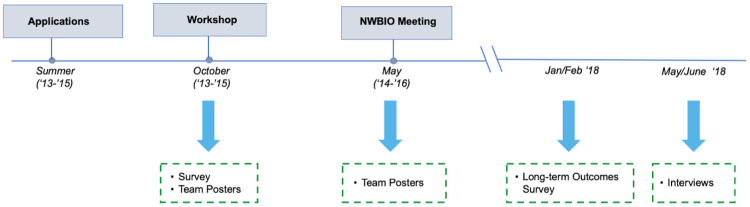
Timeline for collection of data from the first three cohorts.

The NW PULSE professional development activities were offered for five years as part of a grant-funded project (2013–2018). In 2018 an independent external evaluation was conducted (G.F. and C.L.) to (1) better understand the effect NW PULSE had on the participating institutions in the first three institutional cohorts and (2) identify what strategies institutions in the first three cohorts used to transform their approach to undergraduate education in the life sciences. Only the first three cohorts were a focus of the evaluation because it was more likely that sufficient time had passed for these cohorts to initiate departmental transformation. Data for the external evaluation included data that were collected from cohorts 1–3 (2013–2015) as part of their routine participation in the professional development activities (i.e., surveys administered after the October workshops, posters from the workshop and follow-up meeting in May). An additional long-term outcomes survey was administered in 2018 to all teams from cohorts 1–3 to document long-term effects of workshop participation. The timeline for data collection is summarized in Fig 4. The Washington State University Humans Subjects Research Institutional Review Board and the Education Development Center Institutional Review Board reviewed the data collection and analysis protocols and determined they met the criteria for exemption (Exempt—45 CFR 46.101 (b)(2)).

#### Posters

**Fig 5:**
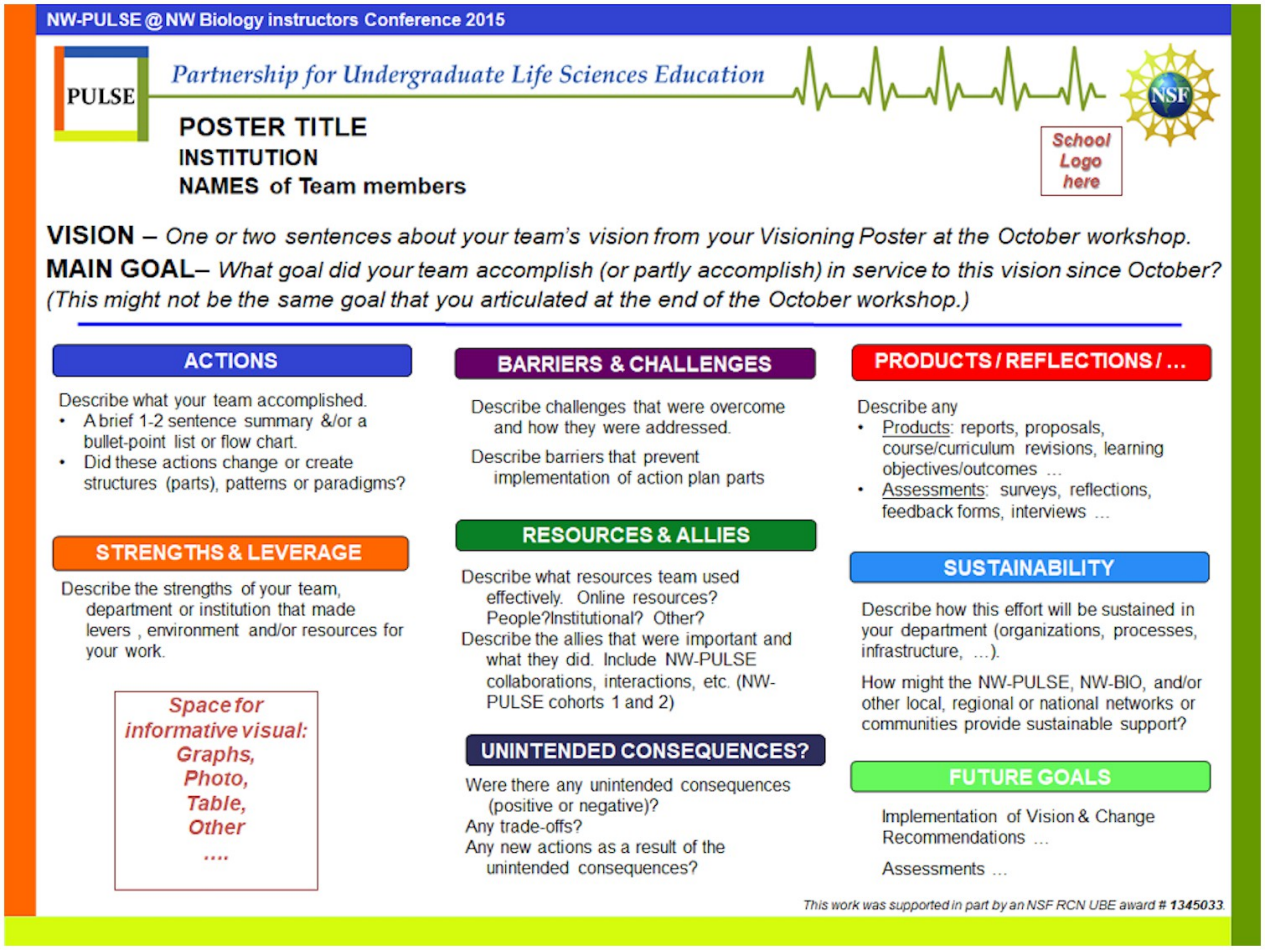
The poster template used for the reporting of progress towards departmental transformation at the May NW BIO conference.

Each institutional team generated two posters during the NW PULSE professional development experience. The first was created during the October workshop to communicate the vision for their department that they developed through the workshop. The second was generated for the networking event at NW BIO regional conference to report the team’s progress-to-date in departmental transformation. A poster template (Fig 5) was provided to each institutional team for the second poster. The “activities” and “outputs” sections of the posters were subsequently analyzed by the independent external evaluators (G.F. and C.L.) using qualitative analysis software Dedoose (www.dedoose.com). The analysis began with an initial reading of the posters, identification of specific text segments or images related to the evaluation objectives, labeling of segments to create categories, and iterative inspection and refinement of categories to identify an emergent coding framework that described actions, accomplishments, barriers and challenges, solutions, and outcomes [19]. G.F. and C.L. ensured trustworthiness of the data analysis by employing routine coding consistency checks including independent parallel coding, category clarification checks, and stakeholder checks [19].

#### Surveys

A survey designed by the PULSE leadership team was electronically distributed to participants approximately seven months after the completion of the workshop. The survey consisted of demographic questions to document institutional and department characteristics (i.e., department size, institution type), Likert-scale questions to determine participants’ perceptions of and interactions within the NW PULSE community, and open-ended questions about the participants’ experiences with transformation since completion of the October workshop. Prior to administration, the survey was reviewed by the NW PULSE External Advisory Board [C.P., S.S., M.P.W.] for face validity [20]. Individual responses to the open-ended questions were qualitatively analyzed by the external evaluators (G.F. and C.L) for themes using the coding framework applied to the posters.

A separate, long-term outcomes survey was developed and distributed in 2018 by the external evaluators (G.F. and C.L.) to document Cohort 1–3 participants’ subsequent progress in department transformation, the strategies employed to enact change, and reflections on NW PULSE’s contribution to departmental reform efforts. Initial drafts of the survey were created by the external evaluators (G.F. and C.L.), reviewed by the PULSE leadership team for readability, alignment with the goals of the evaluation, and as a validity check [20]. The survey (S1 Appendix) was made available to the first three cohorts for one month. Of 138 workshop participants in these cohorts, 79 (57%) completed the survey. We received at least one individual survey response from 39 of the 45 institutions from Cohorts 1–3 (87% response rate). The survey results presented below are at the individual level rather than the departmental level (i.e., percentages represent the total number of *individuals* who selected a survey response and not the percentage of *institutions* that selected a response).

## Results

The overarching goal of this study was to explore the effects of a professional development experience with an explicit focus on systems thinking on departmental transformation efforts in undergraduate biology. To this end, we first document the effect of participation in the NW PULSE workshop on participants’ knowledge about systems thinking and their self-reported use of systems thinking skills after the workshop. We then present evidence that participants applied systems thinking in their change efforts by examining the actions they report applying to initiate department-level transformation. Finally, we report on the participants’ perceptions of success in initiating transformation.

### RQ#1. Did the NW PULSE workshops increase participants’ systems thinking skills?

**Fig 6:**
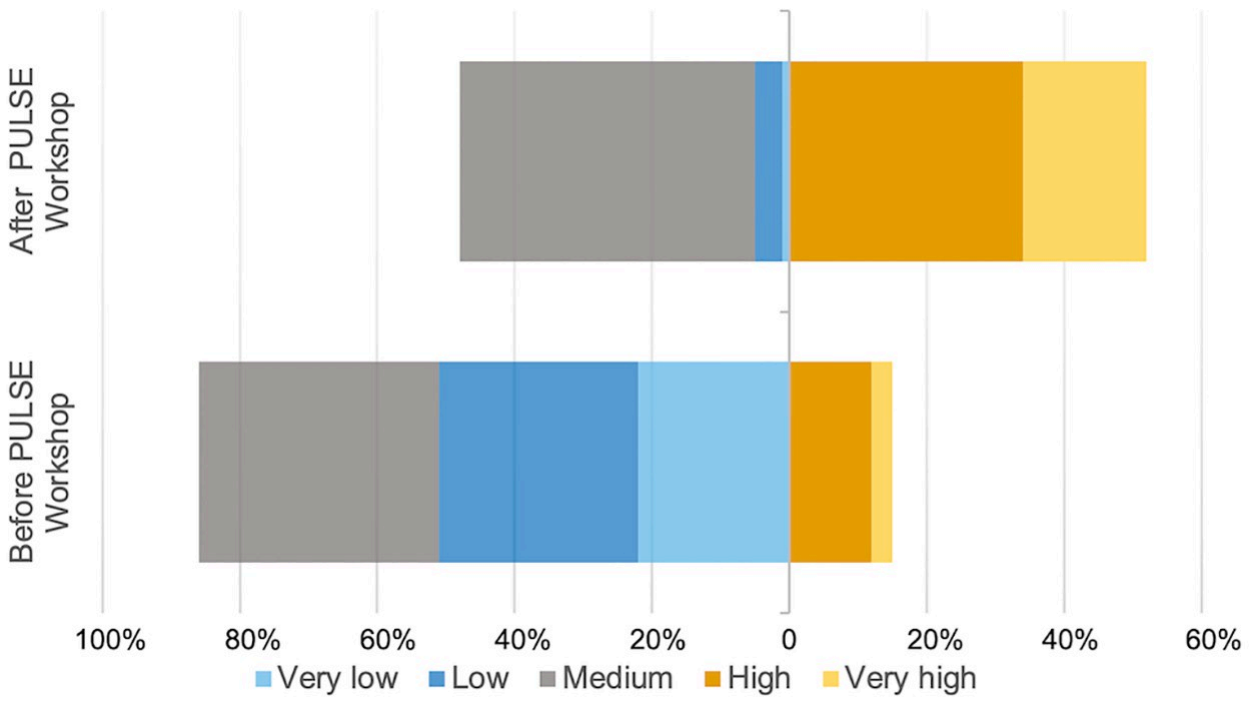
Change in participants’ perceived knowledge of systems thinking. Percentage of participants (n = 77) reporting very low, low, medium, high or very high levels of systems thinking. The percentage of participants reporting high or very high levels of knowledge (orange) increased from before the PULSE workshop (bottom bar) to after the workshop (top bar, (p<0.001, df = 4)).

Participants’ perceived knowledge about systems thinking was measured on the long- term outcomes survey by a 5-level Likert-scale item, “Please consider how your knowledge about systems thinking has changed based on your experience with NW PULSE. Indicate your level of knowledge of systems thinking BEFORE NW PULSE and NOW (following NW PULSE).” Notably, only 15% of participants indicated high or very high knowledge of systems thinking prior to the NW PULSE workshop (Fig 6, lower bar, orange region); after NW PULSE this number increased to 52% (upper bar, orange region). Twenty percent of respondents reported no change in their knowledge, whereas 50% increased by one level and 30% changed by 2 or more levels. A chi-square test of independence confirms a statistically significant increase in participants’ perceived knowledge about systems thinking (p<0.001, df = 4).

### RQ#2. Did NW PULSE participants demonstrate systems thinking when initiating departmental transformation?

**Fig 7:**
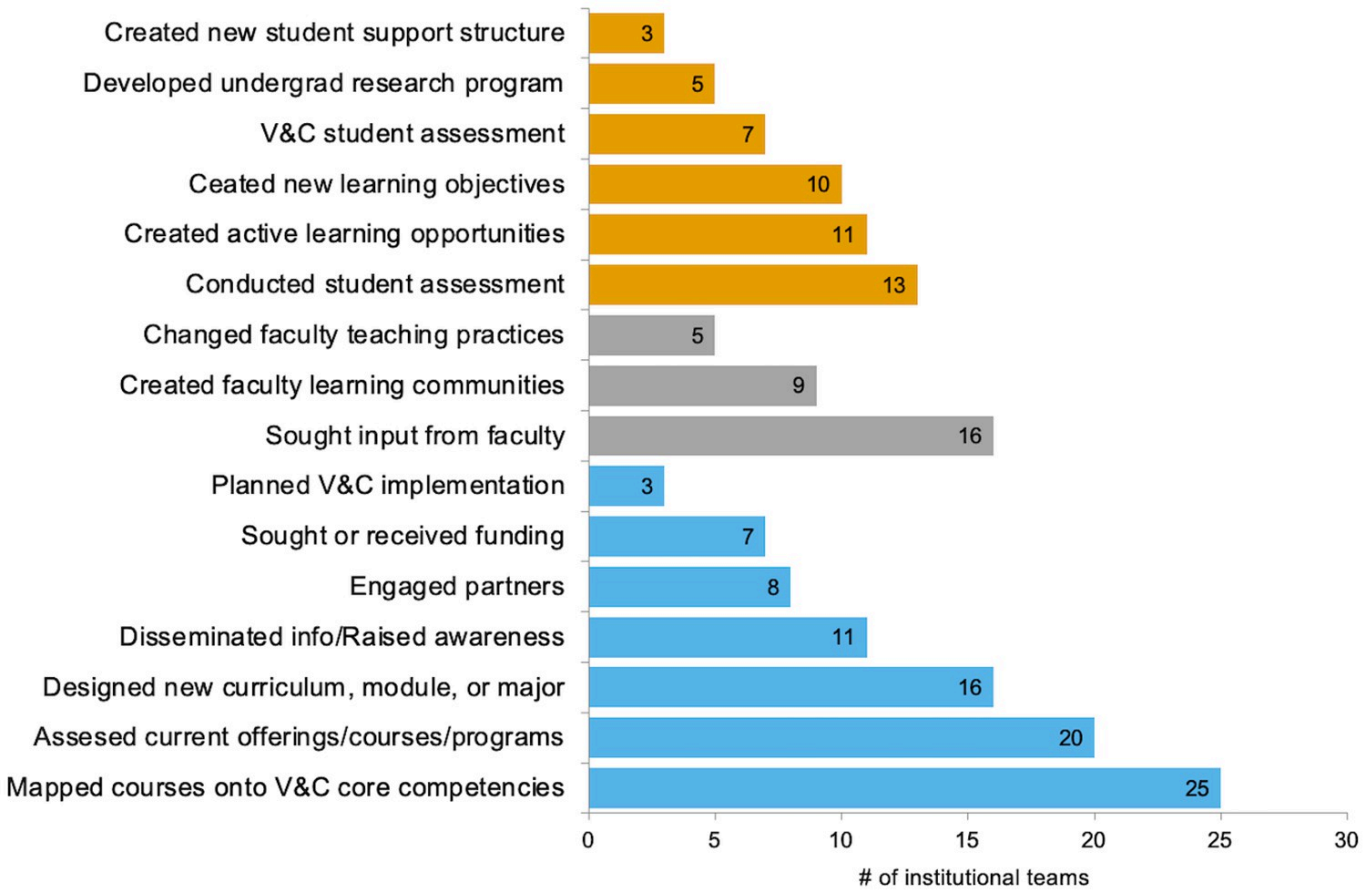
Analysis of progress-to-date posters presented by institutional teams from cohorts 1–3 at NW BIO (n = 42). Sixteen actions at three levels of influence: student (orange), faculty (grey), and department/institution (blue) were identified by two or more institutional teams.

Institutional teams presented their initial transformation efforts following the NW PULSE workshop on their progress-to-date posters at NW BIO. The initial actions taken by institutional teams were focused at three levels of influence: department/institutional level, faculty level, and student level (Fig 7). All teams applied more than one action in their initial transformation efforts.

PULSE professional development workshops were designed for faculty members from all institution types. We were curious whether there were noticeable differences in the types of actions initiated by faculty from different institution type. The most common actions targeted across institution type were at the department/institutional level (blue, Table 1). When viewed through the Four Frames, the majority of initial actions at all institutions were structural changes (e.g., designing curricula, course mapping).

**Table 1:** Actions taken by institution type across cohorts 1–3 as reported on posters at NBIO (n = 40 posters with action data). Each action was reported by at least two different schools. (blue = dept./institution level, grey = faculty level, orange = student level).

Institution Type	Most common actions and # (%) of schools within institution type
Community College (n = 18)	Design new curriculum; 9 (50%)
	Mapping courses to V&C; 9 (50%)
Liberal Arts (n = 8)	Assess current courses; 5 (63%)
	Mapping courses to V&C; 5 (71%)
	New student goals; 4 (50%)
Regional Comprehensive (n = 7)	Mapping courses to V&C; 6 (86%)
	Assess current courses; 4 (57%)
Research-Intensive (n = 7)	Assess current courses; 8 (86%)
	Mapping courses to V&C; 5 (71%)
	Seek faculty input; 5 (71%)

**Fig 8:**
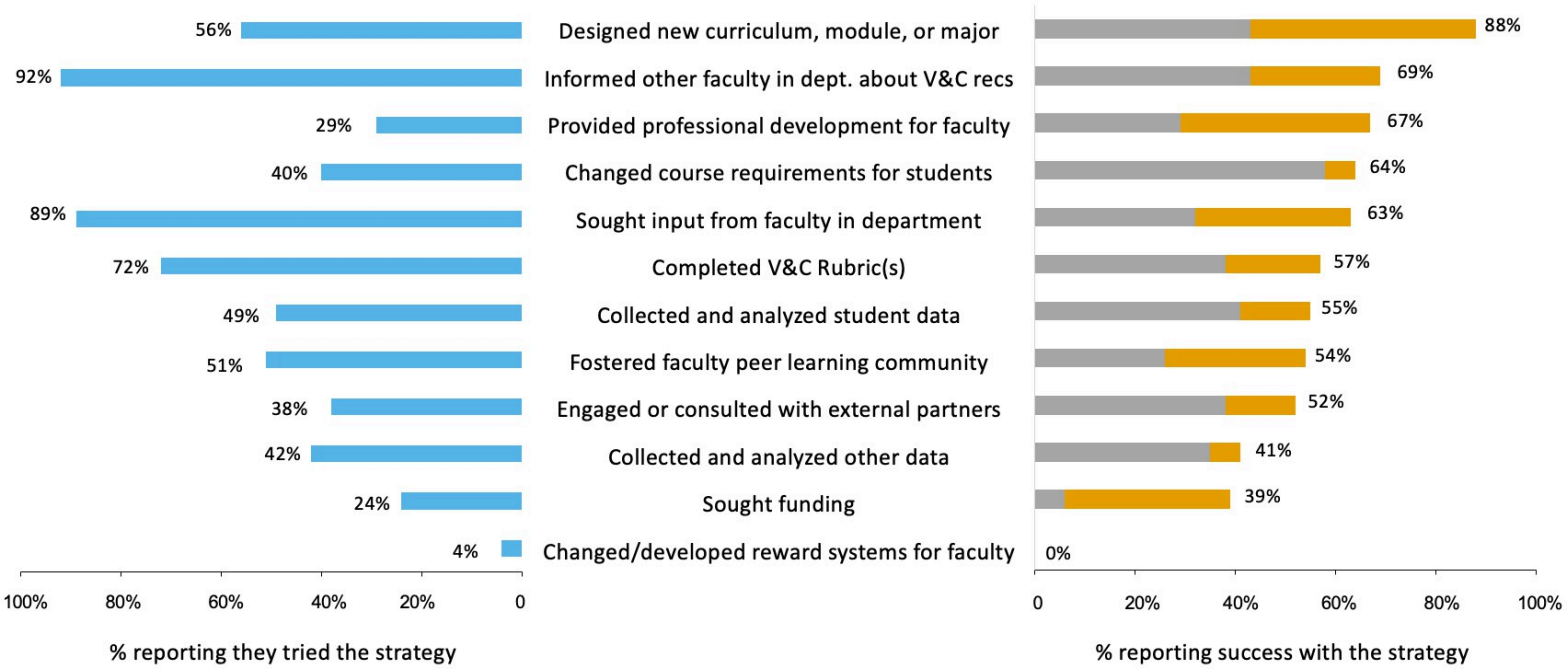
Change strategies tried by participants and reported success of use. Participants (n = 79) were given a list of transformation strategies and asked if they had tried the strategy (left panel) and if they agreed that the strategy was somewhat successful or very successful in helping the team achieve its desired outcome (right panel, grey and orange respectively).

The long-term outcomes survey further documented the actions of institutional teams in their departmental transformation efforts years after participation in the NW PULSE workshop. The survey supplied a table of strategies and asked participants to indicate whether they tried each strategy to achieve their desired outcomes (Fig 8, left panel). Consistent with a systems approach almost all respondents indicated they had tried multiple strategies; teams reported using an average of six actions in their transformation efforts. The two most common strategies (informing faculty, seeking faculty input) were at the department/institutional level, but leverage the human resources frame, which was a departure from initial efforts that leveraged through the structural frame. The two strategies reported as most helpful (Fig 8, right panel) were also at the department/institutional level, but represent two separate frames (structural and human resources).

**Fig 9:**
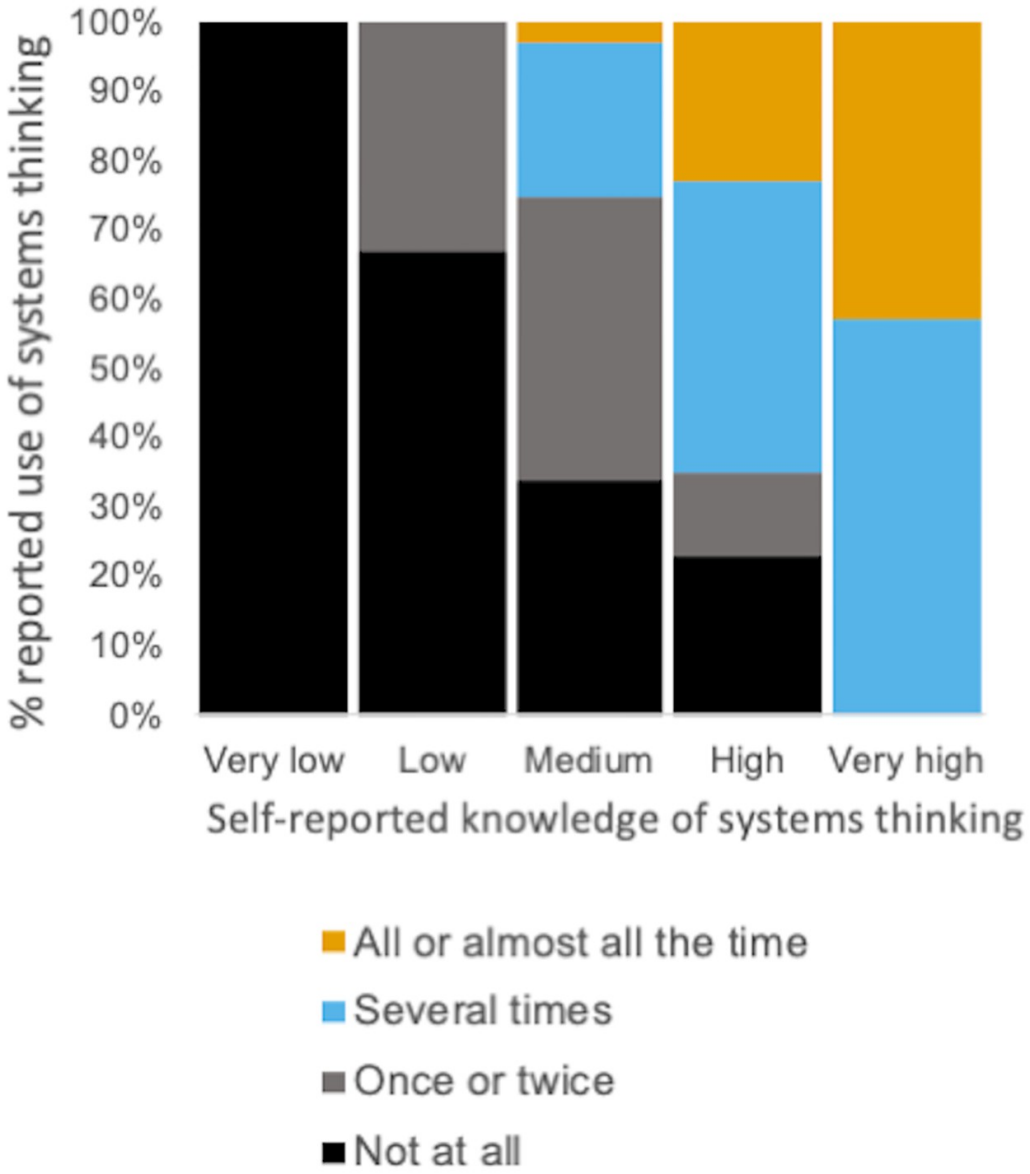
Years after participation, self-reported use of systems thinking is positively correlated with self-reported knowledge about systems thinking. Responses (n = 76) to the Likert-scale question on the long-term outcomes survey, *“How often have you used systems thinking concepts that you learned about through NW PULSE in efforts to move your department towards the recommendations in the Vision and Change report*?*”* are displayed as a function of self-reported knowledge about systems thinking (x-axis).

The long-term outcomes survey also asked participants explicitly about the frequency with which they used systems thinking concepts to promote departmental transformation. Seventy-three percent of responding participants indicated that they used systems thinking at least once after participating in NW PULSE with 51% reporting that they used systems thinking concepts several times or most of the time. Participants reporting “high” or “very high” knowledge about systems thinking after their engagement with NW PULSE most commonly reported using systems thinking concepts “several times” or “all or almost all the time” (Fig 9). These data suggest that as participants self-reported knowledge grew so did the likelihood that they would apply it regularly as part of the execution of their transformation plans (Fig 9).

There were similarities of the most frequently applied actions. Analysis of individual participants’ responses to the open-ended long-term survey prompt “describe what systems thinking concepts you have used and how” demonstrate that, for at least some, certain actions (e.g., redesign curricula) often went hand-in-hand with other actions (e.g., using the PULSE V&C rubrics, seeking faculty input, collecting data):

*“The rubrics were helpful in our faculty identifying priorities for aligning our curriculum with V&C*. *We were successful in making some changes to our labs that allowed for more student inquiry and greater quantitative analysis*. *We have seen learning improvements in these areas*.*”*

*(Survey respondent)*

*“Vision & Change became the guiding document for us as we restructured our curriculum and also as we developed lab courses for non-majors*. *We had full buy-in from the members of the department*. *The conversations about curriculum and the development of peer learning communities have been extremely rewarding and productive*. *Faculty continue to meet routinely with others teaching in their area and the department is now aware of what is being taught in most of its courses—this helps to reinforce and build on the central themes throughout the curriculum*. *Our assessment strategies have greatly improved as well*, *and our curriculum development process has been used as an example at our university of ‘closing the assessment loop*.*’”*

*(Survey respondent)*

When examining these efforts through the Four Frames, actions such as restructuring or redesigning a curriculum would align with approaching departmental transformation through a structural frame. Application of the power frame might lead to the use of data from the rubrics to engender faculty buy-in within the department. These efforts are consistent with a systems approach to organizational change because they seek to exert influence through multiple organizational frames. Similarly, as evidenced by the quote below, some participants expressly identified the importance of considering multiple levels of influence when identifying levers for change, another habit of a systems thinker [13].

*“I really look for levers—how does what I want for my program/students fit with what is good for the University*, *and who would be most effective at advocating for it*.*”*

Collectively, these data suggest that NW PULSE participants not only reported increases in systems thinking knowledge but that several also demonstrated the ability to apply systems thinking to initiate departmental transformation. This was evidenced most prominently in data from the long-term outcomes survey where participants reported applying many actions that targeted various institutional levels and through multiple organizational frames.

### RQ#3. Were the transformation efforts of the NW PULSE participants perceived to be successful?

**Fig 10:**
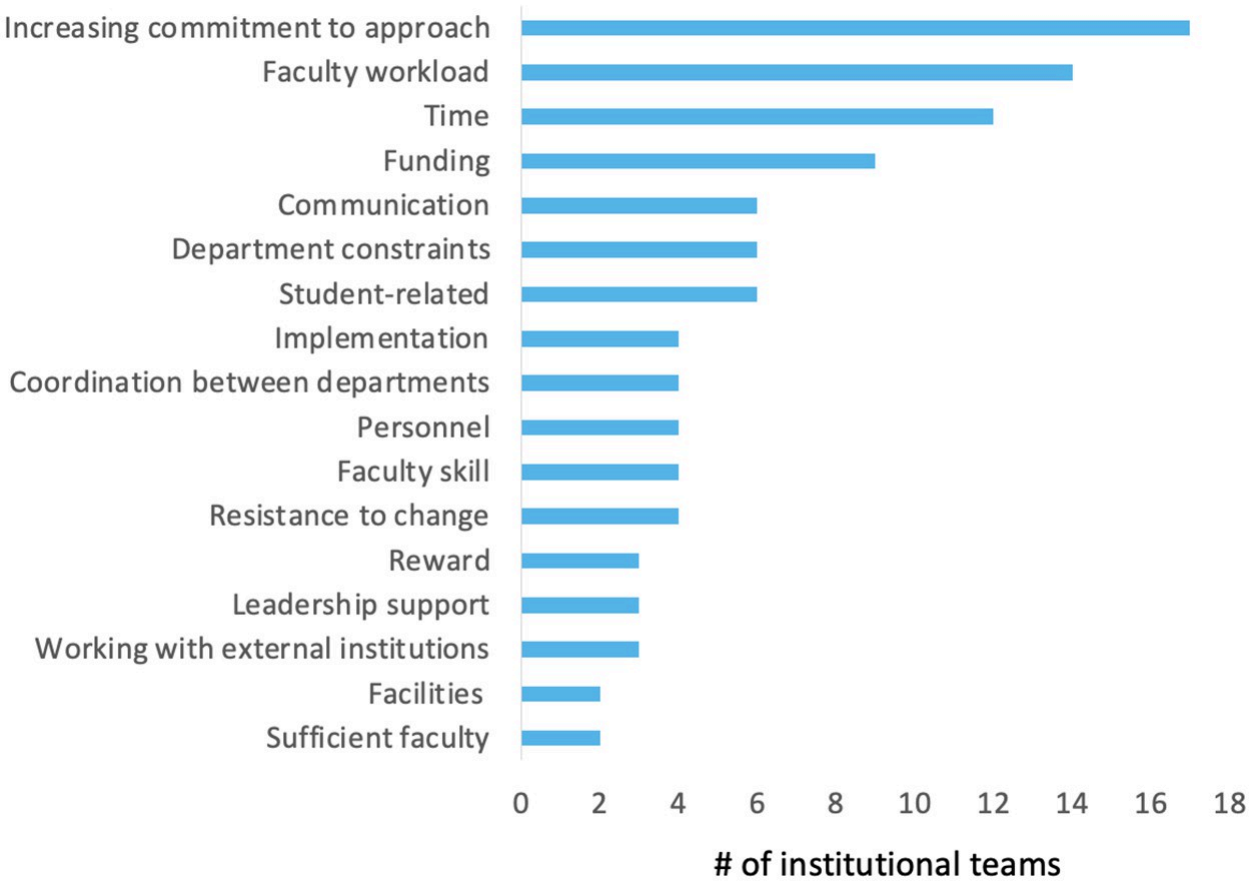
Barriers identified on progress-to-date posters presented at the NW BIO conference (n = 36 posters with barriers data).

Institutional teams reported 37 different barriers, including 27 that were shared among at least two schools and 10 that were unique to a single institution (Fig 10). The most frequently cited barriers were related to increasing faculty commitment to an approach (Fig 10), with 17 groups (47%) mentioning the challenge of raising awareness of the NW PULSE project and their action plans or getting faculty involvement or buy-in. The second and third most frequently cited barriers were associated with time. Fourteen groups (39%) provided responses related to faculty workload, including time to dedicate to the effort, and 12 groups (33%) indicated that they struggled with having time to plan, execute and see Results. Barriers varied considerably by individual institution, with no barriers emerging as experienced by the majority of institutions.

Although the frequency with which respondents from different institutional types cited particular barriers varied somewhat, similar challenges were encountered across institutional types (e.g., the need to increase awareness/buy-in, Table 2).

**Table 2:** Most common challenges reported by institutions on their progress-to-date posters presented at NW BIO reported by institution type (n = 36 of 40 posters reported challenges). Barriers are matched by color if they appeared in more than one institution type.

Institution Type	Most common challenges and # (%) of schools
Community College (n = 18)	Staff workload; 8 (47%)
	Increasing awareness/buy-in; 7 (41%)
	Funding; 7 (41%)
Liberal Arts (n = 8)	Increasing awareness/buy-in; 4 (57%)
	Funding, 2 (29%)
	Implementing plan; 2 (29%)
	Time to execute and see Results; 2 (29%)
Regional Comprehensive (n = 7)	Staff workload, 8 (47%)
	Increasing awareness/buy-in; 4 (67%)
Research-Intensive (n = 7)	Time to execute and see Results; 5 (83%)
	Increasing awareness/buy-in; 2 (33%)

The long-term outcomes survey asked respondents to reflect on the degree to which each of the actions they employed were successful (Fig 8, right panel). While participants indicated that some efforts were more successful than others, most of the actions used by participants were at least somewhat successful. Modifying the curriculum was one of the most frequently used strategies, and also among the most successful. More than half the survey respondents (56%) reported that they had designed a new curriculum, module or major, and the vast majority (88%) of those who had done so reported that it was at least “somewhat” successful. A smaller percentage of survey respondents (40%) reported that their institutions had changed course requirements for students, with 64% of those who had done so indicating it was at least somewhat successful.

## Discussion

The NW PULSE leadership developed regional professional development experiences to facilitate department-level changes in the life sciences in the Northwestern US, an effort in line with national initiatives on science education [4, 8]. The experience was designed with an explicit focus on developing participants’ knowledge of systems thinking as an approach for organizational change [13]. The application of systems thinking to identify areas for improvement using the Four Frames model was a novel approach, as was our attempt to identify whether this effort was effective in catalyzing transformation. We present summary self-reported data that systems thinking is an effective strategy to help faculty and administrators critically examine departmental practices, identify areas for improvement, and undertake efforts to transform the academic experience of students in life sciences.

Institutional teams consisting of life sciences faculty and administrators participated in a sustained professional development experience that included a three-day workshop, ongoing mentoring, and culminating networking event. After completing the three-day workshop, participants reported an increase in knowledge of systems thinking skills (Fig 6). This self- reported increase in knowledge was positively correlated with participant reports of using systems thinking upon returning to their home institutions years after the initial workshop experience (Fig 9). It is possible that participants already possessed systems thinking skills prior to workshop participation but simply did not have the vocabulary for formally describing their skills as “systems thinking”. Therefore, some of the increase in self-reported knowledge of systems thinking may simply be a consequence of providing a vocabulary and framework to describe some habits of mind already possessed by some of our participants. Nevertheless, participants who reported higher levels of knowledge were more likely to also report using systems thinking in their transformation effort. At a minimum, it is reasonable to conclude that the intentional focus on introducing systems thinking did result in participants who were able to identify and implement a systems thinking approach. We argue that raising awareness of systems thinking as an approach for departmental transformation is a valuable first step in empowering future faculty members to devise change approaches that work for their individual departmental context.

The organizational change literature indicates that transformation efforts that apply multiple strategies are consistent with a systems approach, as are efforts that leverage change through multiple organizational frames [7, 9, 17, 18]. When we reflected on the external evaluation data, these were the two criteria we used to determine whether participants demonstrated systems thinking in their transformation efforts. Teams leveraged an average of six multiple actions in their transformation efforts. Initially the most common actions targeted structural changes at the department/institutional level (Fig 7, Table 1). As measured on the long-term outcomes survey, the most common actions reported by teams were at the faculty level and sought to leverage change through human resources frame (Fig 8). Participants who reported using systems thinking upon returning to their home institutions were also more likely to report initial successes in their department transformation efforts. While the length of time between initial PULSE work participation and completion of the long-term outcomes survey was different among cohorts, participant reports of multiple strategies at different levels and through more than one frame is evidence that teams were applying some systems thinking concepts. Given the often-lethargic rate of change in higher education settings, future research should more carefully examine the ways in which transformation strategies must evolve over time and in response to department/institutional context.

As other authors have described, context plays a significant role in the potential success of transformation efforts in STEM education [21, 22]. The barriers to change faced by NW PULSE participants were similar to those identified in other STEM education transformation projects [22]. The self-reported data presented in this report were collected from participants representing a variety of institution types. This diversity of voices was important, as it was reflective of both the composition of the NW PULSE leadership group, but also of the multiple institutional paths American students follow to complete their undergraduate education. Interestingly, we identified barriers shared among institution types, and that the initial strategies used to instigate transformation were similar. The small number of schools within each subgroup is a limitation of the study, so these findings should be considered with caution, but it appears that the principal strategies for transformation and the barriers that impede them are common to all institution types that participated in our workshops. The efficacy of systems thinking for departmental transformation needs to be further validated with a greater number of departments from a variety of institution types and STEM disciplines. However, our data suggest that systems thinking is an accessible and beneficial method for faculty to identify common levers for overcoming the barriers that manifest within different contexts. While there is no “one-size-fits-all” approach to department-level change, we argue that systems thinking is a useful tool that can facilitate a contextually responsive approach to change initiatives.

Together our data provide some reason to be optimistic regarding the utility of systems thinking as one approach to support life sciences faculty in adopting the large-scale changes recommended in V&C. Moreover, while this experience was designed to support life sciences faculty, departments in other STEM discipline are likely to share some structural and cultural similarities. Therefore, faculty across STEM disciplines may benefit from learning about systems thinking as it relates to effecting change.

## Conclusion and future directions

To our knowledge, this is the first empirical study investigating whether training in systems thinking has a positive effect on department-level transformation efforts in the life sciences. The data presented here suggest that professional development opportunities that focus on systems thinking increase faculty members’ knowledge and abilities to apply a systems approach to department-level change. Further, it appears that life science educators and administrators extended their work beyond training in systems thinking to identifying and addressing departmental transformation. The tentative evidence presented in support of this assertion is important since participants did not simply follow a prescribed strategy over and over, but their choices demonstrate a systems thinking approach using multiple frames. Additional work should seek to understand the degree to which other professional development or other experiences could have contributed to faculty applying a systems approach. Immediate next steps would be to continue to follow NW PULSE participants in their efforts over time and to collect departmental documents and artifacts (e.g., curriculum maps, promotion and tenure criteria, teaching loads) to characterize the nature and extent of transformation. We need additional evidence to corroborate self-report data and to better understand if and how the various strategies for transformation change over time as teams encounter obstacles and identify levers.

Finally, this work raises questions about the necessary conditions for comprehensive departmental transformation. Although not all participants reported the same levels of success, the results presented here suggest that application of systems thinking can improve the prospects for success of change initiatives. Our data suggest that training on systems thinking is useful for faculty to initiate transformation, yet future research might seek to understand how other skills or knowledge (e.g., leadership skills, skills in navigating interpersonal conflict, knowledge of other principles of organizational change) would complement a systems thinking approach to increase the likelihood of long-lasting institutional transformation.

## Supporting information

S1 Appendix Long-term outcomes survey. (PDF)

## References

[1] Brewer CA, Smith D. Vision and change in undergraduate biology education: a call to action. American Association for the Advancement of Science, Washington, DC. 2011 Feb;81.

[2] National Institute on Scientific Teaching [Internet]. [cited 2021 Sept 15] https://www.nisthub.org

[3] Association of American Universities [Internet]. Washington: Association of American Universities [c2021] [cited 2021 Sept 15] https://www.aau.edu/sites/default/files/AAU-Files/STEM-Education-Initiative/AAU-STEM-Essential_Questions.pdf

[4] Beach AL, Henderson C, Finkelstein N. Facilitating change in undergraduate STEM education. Change: The Magazine of Higher Learning. 2012 Nov 1;44(6):52–9.

[5] Peteroy-Kelly M, Brancaccio-Taras L, Awong-Taylor J, Balser T, Jack T, Lindsay S, et al. A qualitative analysis to identify the elements that support department level change in the life sciences: The PULSE Vision & Change Recognition Program. Plos one. 2019 May 30;14(5):e0217088. doi: 10.1371/journal.pone.0217088 31145735 PMC6542552

[6] Brancaccio-Taras L, Pape-Lindstrom P, Peteroy-Kelly M, Aguirre K, Awong-Taylor J, Balser T, et al. The PULSE Vision & Change Rubrics, Version 1.0: A valid and equitable tool to measure transformation of life sciences departments at all institution types. CBE—Life Sciences Education. 2016 Dec;15(4):ar60. doi: 10.1187/cbe.15-12-0260 27856548 PMC5132357

[7] Austin AE. Promoting evidence-based change in undergraduate science education. In Fourth committee meeting on status, contributions, and future directions of discipline- based education research. 2011 Mar 1.

[8] DeMarais A, Bangera G, Bronson C, Byers S, Davis W, Linder N, et al. What Lies Beneath? A Systems Thinking Approach to Catalyzing Department-Level Curricular and Pedagogical Reform Through the Northwest PULSE Workshops. Transformative Dialogues: Teaching and Learning Journal. 2022 Mar 7;14(3).

[9] Aguirre KM, Balser TC, Jack T, Marley KE, Miller KG, Osgood MP, et al. PULSE vision & change rubrics. CBE—Life Sciences Education. 2013 Dec;12(4):579–81. doi: 10.1187/cbe.13-09-0183 24297283 PMC3846506

[10] Bolman LG, Deal TE. Reframing organizations: Artistry, choice, and leadership. John Wiley & Sons; 2017 Jul 24.

[11] Henderson C, Beach A, Finkelstein N. Facilitating change in undergraduate STEM instructional practices: An analytic review of the literature. Journal of Research in Science Teaching. 2011 Oct;48(8):952–84.

[12] Manning K. Organizational theory in higher education. Routledge; 2017 Sep 14.

[13] Senge PM. The fifth discipline: The art and practice of the learning organization. Currency; 2006.

[14] Kezar A. Change in higher education: Not enough, or too much? Change: The Magazine of Higher Learning. 2009 Sep 30;41(6):18–23.

[15] Kezar A. Departmental cultures and non-tenure-track faculty: Willingness, capacity, and opportunity to perform at four-year institutions. The Journal of Higher Education. 2013 Mar 1;84(2):153–88.

[16] Senge PM, Cambron-McCabe N, Lucas T, Smith B, Dutton J. Schools that learn (updated and revised): A fifth discipline fieldbook for educators, parents, and everyone who cares about education. Currency; 2012 Jul 31.

[17] Reinholz DL, Apkarian N. Four frames for systemic change in STEM departments. International Journal of STEM Education. 2018 Dec;5(1):1–10. doi: 10.1186/s40594-018-0103-x 30631693 PMC6310387

[18] Reinholz DL, Ngai C, Quan G, Pilgrim ME, Corbo JC, Finkelstein N. Fostering sustainable improvements in science education: An analysis through four frames. Science Education. 2019 Sep;103(5):1125–50.

[19] Thomas DR. A general inductive approach for analyzing qualitative evaluation data. American Journal of Evaluation. 2006 Jun;27(2):237–46.

[20] Patton MQ. Qualitative research & evaluation methods: Integrating theory and practice. Sage publications; 2014 Oct 29.

[21] Lund TJ, Stains M. The importance of context: an exploration of factors influencing the adoption of student-centered teaching among chemistry, biology, and physics faculty. International Journal of STEM Education. 2015 Dec;2(1):1–21.

[22] Shadle SE, Marker A, Earl B. Faculty drivers and barriers: laying the groundwork for undergraduate STEM education reform in academic departments. International Journal of STEM Education. 2017 Dec;4(1):1–3. doi: 10.1186/s40594-017-0062-7 30631664 PMC6310369

