# Supplementary material for: Empowering faculty to initiate STEM education transformation: Efficacy of a systems thinking approach
Source: PLoS One. 2022 Jul 25;17(7):e0271123. doi: 10.1371/journal.pone.0271123 (PMC9312400; doi:10.1371/journal.pone.0271123)
Supplement: S1 Appendix — (PDF) [file pone.0271123.s001.pdf]

NW PULSE Follow Up Survey

NW PULSE Follow-Up Survey

As a past participant of the NW PULSE project, you are being asked to complete a survey about your experiences in the project. The survey will take about 15-20 minutes.

While we expect that you can answer most of the questions from memory, it may be helpful to have your V&C assessment rubric(s) available to further your recollection and help you answer a few questions (to access your school's V&C files, open the following link in a new tab on your browser: http://www.pulsecommunity.org/group/washington-state-v-c-fellow/page/2014-nwbio-posters-from-nw-pulse-community-of-practice-members.   There are no right or wrong answers to the questions to the questions on this survey; we are looking for an honest assessment of how your involvement in NW PULSE has influenced your department and/or institution.

Your responses are kept confidential by the evaluation team administering this survey. We aggregate data to report findings to the NW PULSE project team and remove identifying information from quotes.

Thank you for taking the time to help the NW PULSE project! Please contact us with any questions.

Sincerely,

Ginger Fitzhugh and Carrie Liston
Education Development Center
gfitzhugh@edc.org
206-395-4528

Background Question

What is your position at your institution (e.g., Professor, Assistant Professor, Dean)?

____

____

____

____

____

NW PULSE Components

Please indicate how helpful each of the following NW PULSE components was to you.
Select N/A if you did not participate in that component.

	**N/A** (1)	**Not at all useful** (2)	**Not very useful** (3)	**Neutral** (4)	**Useful** (5)	**Very useful** (6)
PULSE Vision & Change Rubrics (1)	○	○	○	○	○	○
Three-day NW PULSE October workshop (2)	○	○	○	○	○	○
Follow-up support from NW PULSE Coach (3)	○	○	○	○	○	○
Networking with other NW PULSE participants (4)	○	○	○	○	○	○
Resources and other materials on the NW PULSE website (http://www.pulsecommunity.org) (5)	○	○	○	○	○	○
NWBio PULSE Follow up Workshop (6)	○	○	○	○	○	○

Which of the above NW PULSE components were most helpful to you and why?

____

____

____

____

____

For any components that you rated as “Not at all useful” or “Not very useful,” please explain your response and/or add suggestions on what might make the component more useful:

____

____

____

On average, about how often did you or somebody at your institution communicate with your NW PULSE Fellow in the year after your participation in the October workshop?

- ○ Less than once a year (1)
- ○ About once per year (2)
- ○ A few times per year (3)
- ○ Every other month (4)
- ○ About once a month (5)
- ○ Every other week (6)
- ○ Once a week or more (7)

What, if any, additional resources would have been helpful to have to support your work in moving your department towards the recommendations in the Vision and Change Report?

Your Department's Vision and Change Plan

Which area(s) of the Vision and Change recommendations did your department identify as one(s) that you wanted to transform? *(Check all that apply.)*

- ▢ Curriculum alignment (1)
- ▢ Assessment (2)
- ▢ Faculty practice/faculty support (3)
- ▢ Infrastructure (4)
- ▢ Climate for change (5)
- ▢ Other; please describe: (6) ____________________

As part of the October workshop, your team created an action plan to help guide your work at your institution. **To what degree did you implement your action plan?**

- ○ Exactly as planned (1)
- ○ Almost entirely as planned, with a few changes (2)
- ○ Somewhat as planned, but with many changes (3)
- ○ Not at all as planned; we implemented an entirely different plan (4)
- ○ Not at all as planned; we have not implemented any action plans at this time (5)
- ○ N/A: Unable to answer – do not remember our original action plan (6)

The table below lists a number of possible strategies that your NW PULSE team may or may not have identified as potentially useful for achieving your goals at your institution. **Please indicate whether or not your institution tried each strategy.**

	**Yes** (1)	**No** (2)
Completed Vision & Change Rubric(s) (1)	○	○
Informed other faculty in department about Vision & Change recommendations (2)	○	○
Sought input from faculty in department (3)	○	○
Collected and analyzed student data (4)	○	○
Collected and analyzed other data (5)	○	○
Designed new curriculum, module or major (6)	○	○
Changed course requirements for students (7)	○	○
Provided professional development for faculty (8)	○	○
Fostered faculty peer learning community (9)	○	○
Changed or developed reward systems for faculty (10)	○	○
Engaged or consulted with external partners (e.g., from other campuses) (11)	○	○
Sought funding (12)	○	○

Carry Forward Selected Choices from "The table below lists a number of possible strategies that your NW PULSE team may or may not have identified as potentially useful for achieving your goals at your institution. Please indicate whether or not your institution tried each strategy."

For each strategy you have used, indicate the degree to which it has helped your institution achieve desired change. *Drag and drop each strategy from the left into the appropriate box on the right, with the response scale from "Very unsuccessful (1)" to "Very successful (5)".*

Very unsuccessful (1)	Somewhat unsuccessful (2)	Mixed (3)	Somewhat succesful (4)	Very successful (5)
______ Completed Vision & Change Rubric(s) (1)	______ Completed Vision & Change Rubric(s) (1)	______ Completed Vision & Change Rubric(s) (1)	______ Completed Vision & Change Rubric(s) (1)	______ Completed Vision & Change Rubric(s) (1)
______ Informed other faculty in department about Vision & Change recommendations (2)	______ Informed other faculty in department about Vision & Change recommendations (2)	______ Informed other faculty in department about Vision & Change recommendations (2)	______ Informed other faculty in department about Vision & Change recommendations (2)	______ Informed other faculty in department about Vision & Change recommendations (2)
______ Sought input from faculty in department (3)	______ Sought input from faculty in department (3)	______ Sought input from faculty in department (3)	______ Sought input from faculty in department (3)	______ Sought input from faculty in department (3)
______ Collected and analyzed <u>student</u> data (4)	______ Collected and analyzed <u>student</u> data (4)	______ Collected and analyzed <u>student</u> data (4)	______ Collected and analyzed <u>student</u> data (4)	______ Collected and analyzed <u>student</u> data (4)
______ Collected and analyzed <u>other</u> data (5)	______ Collected and analyzed <u>other</u> data (5)	______ Collected and analyzed <u>other</u> data (5)	______ Collected and analyzed <u>other</u> data (5)	______ Collected and analyzed <u>other</u> data (5)
______ Designed new curriculum, module or major (6)	______ Designed new curriculum, module or major (6)	______ Designed new curriculum, module or major (6)	______ Designed new curriculum, module or major (6)	______ Designed new curriculum, module or major (6)
______ Changed course requirements for students (7)	______ Changed course requirements for students (7)	______ Changed course requirements for students (7)	______ Changed course requirements for students (7)	______ Changed course requirements for students (7)
______ Provided professional development for faculty (8)	______ Provided professional development for faculty (8)	______ Provided professional development for faculty (8)	______ Provided professional development for faculty (8)	______ Provided professional development for faculty (8)
______ Fostered faculty peer learning community (9)	______ Fostered faculty peer learning community (9)	______ Fostered faculty peer learning community (9)	______ Fostered faculty peer learning community (9)	______ Fostered faculty peer learning community (9)
______ Changed or developed reward systems for faculty (10)	______ Changed or developed reward systems for faculty (10)	______ Changed or developed reward systems for faculty (10)	______ Changed or developed reward systems for faculty (10)	______ Changed or developed reward systems for faculty (10)
______ Engaged or consulted with external partners (e.g., from other campuses) (11)	______ Engaged or consulted with external partners (e.g., from other campuses) (11)	______ Engaged or consulted with external partners (e.g., from other campuses) (11)	______ Engaged or consulted with external partners (e.g., from other campuses) (11)	______ Engaged or consulted with external partners (e.g., from other campuses) (11)
______ Sought funding (12)	______ Sought funding (12)	______ Sought funding (12)	______ Sought funding (12)	______ Sought funding (12)

For any strategies that you rated as "very" or "somewhat" successful, what made them particularly effective at your institution?

____

____

____

____

____

For any strategies that you rated as "very" or "somewhat" unsuccessful, what made them relatively less effective at your institution?

____

____

____

____

____

If there are other strategies that your team identified and tried, please describe what they were and the degree to which those strategies helped achieve change.

____

____

____

____

____

Did you share anything your learned at the October NW PULSE workshop with colleagues at your institution who did not attend the workshop?

○ Yes (7)

○ No (8)

Display This Question:

If Did you share anything your learned at the October NW PULSE workshop with colleagues at your inst... = Yes

What did you share from the October NW PULSE workshop and who did you share it with at your institution?

____

____

____

____

____

About what proportion of the faculty in your department was involved with the effort to move your department towards the recommendations in the Vision and Change Report? (Note your best estimate as a percentage.)

○ Percent (whole number between 0 and 100): (1) ____________________

Impact of NW PULSE

It's been some time since you participated in NW PULSE. **How much of a difference has your involvement in NW PULSE had in your department?**

- ◯ No difference (1)
- ◯ Small difference (2)
- ◯ Moderate difference (3)
- ◯ Large difference (4)

Please explain your answer.

____

____

____

____

____

What changes, if any, have occurred in your department/institution since you participated in NW PULSE that you attribute to your school's participation in the project?

____

____

____

____

____

Please indicate the degree to which each of the following factors helped or hindered your institution’s efforts to implement your NW PULSE goals.
Select N/A if the factor did not affect your work.

	**N/A; not a factor** (1)	**A very negative factor** (2)	**A somewhat negative factor** (3)	**Mixed; somewhat negative or positive factor** (4)	**A somewhat positive factor** (5)	**A very positive factor** (6)
Senior administration’s (e.g., chair, dean) awareness of department’s change effort arising from NW PULSE engagement (1)	○	○	○	○	○	○
Senior administration’s (e.g., chair, dean) support of department’s change effort arising from NW PULSE engagement (2)	○	○	○	○	○	○
Other faculty’s awareness of department’s change effort arising from NW PULSE engagement (faculty not part of NW PULSE team) (3)	○	○	○	○	○	○
Other faculty’s support of department’s change effort arising from NW PULSE engagement (faculty not part of NW PULSE team) (4)	○	○	○	○	○	○
Time of NW PULSE team members to devote to effort (5)	○	○	○	○	○	○
Time of other faculty in department to devote to effort (6)	○	○	○	○	○	○
Funding (7)	○	○	○	○	○	○
Needs or expectations of students in your department (8)	○	○	○	○	○	○

What do you think made efforts to make changes in your department through NW PULSE particularly successful or unsuccessful?

____

____

____

____

____

Please consider how your knowledge about systems thinking has changed based on your experience with NW PULSE. **Indicate your level of knowledge of systems thinking BEFORE NW PULSE and NOW (following NW PULSE).**

	**Very Low** (1)	**Low** (2)	**Medium** (3)	**High** (4)	**Very High** (5)
Your knowledge about systems thinking concepts **before NW PULSE** (1)	○	○	○	○	○
Your knowledge about systems thinking concepts **now** (following NW PULSE) (2)	○	○	○	○	○

The NW PULSE October workshop introduced a number of systems thinking concepts, including looking at the big picture, considering multiple perspectives and multiple levers for change, and anticipating nonlinear results or unintended consequences. **How often have you used systems thinking concepts that you learned about through NW PULSE in efforts to move your department towards the recommendations in the Vision and Change Report?**

- ○ Not at all (1)
- ○ Once or twice (2)
- ○ Several times (3)
- ○ All or almost all of the time (4)

Display This Question:

If The NW PULSE October workshop introduced a number of systems thinking concepts, including looking... = Once or twice

Or The NW PULSE October workshop introduced a number of systems thinking concepts, including looking... = Several times

Or The NW PULSE October workshop introduced a number of systems thinking concepts, including looking... = All or almost all of the time

Please describe what systems thinking concepts you have used and how.

____

____

____

____

____

Please share other comments or observations you have about NW PULSE and what, if any, difference it has made for your department or institution.

____

____

____

____
